# Romanian Students’ Environment-Related Routines during COVID-19 Home Confinement: Water, Plastic, and Paper Consumption

**DOI:** 10.3390/ijerph18158209

**Published:** 2021-08-03

**Authors:** Vasile Gherheș, Mariana Cernicova-Buca, Marcela Alina Fărcașiu, Adina Palea

**Affiliations:** Department of Communication and Foreign Languages, Politehnica University of Timisoara, 300006 Timisoara, Romania; marcela.farcasiu@upt.ro (M.A.F.); Adina.palea@upt.ro (A.P.)

**Keywords:** student campus, post-COVID-19 higher education, socio-ecological system, territorial sustainability, environmental policy, green university, environment-related routine, water consumption, plastic consumption, paper consumption

## Abstract

The disruptive force of the COVID-19 pandemic is lessening in power and plans are being made for the postcrisis period, among which increasing the sustainability of higher education is of significant importance. The study aims at establishing students’ existing environment-related routines during their home confinement, as a basis for applying green measures to campus living once academic life is resumed with the physical presence of students. The collected data rely on self-reported information provided by 816 students from Politehnica University of Timisoara (Romania), collected via an online, anonymous survey. The novelty of the approach is that household environment-related routines are investigated during a crisis period, with the possibility to build upon the results to implement tailored measures to encourage or diminish environmentally relevant consumption by young, highly skilled individuals. The students display a moderate awareness of environmental issues and indicate consumption routines that may be steered towards an increased sustainability-conscious campus life, through the combined intervention of the university, city administration, and stakeholder involvement. The findings are used to explore the possible directions for action towards increasing or contributing to the territorial sustainability in the socio-ecological context of Timisoara, the largest university city in the western part of Romania via educational, managerial and policy interventions.

## 1. Introduction

Students’ daily routines changed dramatically with the outbreak of the COVID-19 pandemic. Instead of commuting to college, meeting with peers, drinking morning and break coffee, bringing along plastic bottles of water or refreshing drinks to school, taking notes on paper and/or handing in home assignments, final projects or exam work on paper, they had to stay indoors, many of them—instead of in campus dormitories—in their parents’ homes, drastically limit face-to-face interactions and outdoor activities, adopt social distancing rules, use digitally supported solutions for their school life and other activities, and submit electronic versions of their academic-related work. The impact of the pandemic on students’ lives is a topic of great interest, analyzed from a variety of angles, such as the resilience of students and their capacity to continue their professional trajectories [1], the existence of digital infrastructure and the (digital) habits for solving academic and life-related issues, or the students’ satisfaction with the support offered by universities [2]. Equally important are research items that investigate the probability that the post-pandemic life is a digitally more inclusive one [3,4] and, at the same time, a more sustainable, environmentally conscious one [5,6], where young people are expected to display a solid range of sustainability competences, from green lifestyles and green economy to circular economy and environmentally friendly mindsets [7,8].

Prior to the COVID-19 pandemic outbreak in 2020, an event that confined to their homes more than 1.37 billion students of all levels of education, according to the statistics provided by UNESCO [9], studies on students’ environment-related behaviors dealt with either their behavior on campus [10], at college or as part of their generation, in the household, from multiple angles—as part of a lifestyle [11], as a consciously adopted choice [12], as perceptions related to sustainability actions in green universities [10], up to behaviors and attitudes on campus [13,14]. Universities also attracted their share of attention, mainly in the form of calls to action under the framework of the United Nations Environment Programme for going green and joining the Green Universities Network (UNEP) [15], or as practices developed on regional or local levels [10,16,17,18]. The health crisis interrupted much of the habitual academic life and forced universities to focus on their capacity to deliver education and reduce the disruptive force of the pandemic on academic life, by accelerating the adoption of digitalization [19], a topic that was viewed mainly from a technological angle and with an impact on educational practice, but that has a spillover effect on the environmental concerns and greening, if the reduction of physical paper is taken under consideration.

If post-COVID-19 higher education is to be oriented towards adopting environmental innovations and steered towards increasing the sustainability commitment, as part of the action plan to increase the resilience of the university as the main organization relevant for preparing a highly skilled labor force and serving as beacon of knowledge, a profound rethinking of the *modus operandi* is necessary [20]. Additionally, it is important to keep in mind that, while universities can be viewed as agents of change and drivers of innovation in the regions where they operate, their activity is not isolated from the socio-economic, administrative, and political context of the territory [21,22], and their large-scale decisions are intertwined with the capabilities and prerequisites existing in that given territory [10,15]. Therefore, future planning at the university level should resonate and influence, wherever possible, the public policies in the geographical space where they operate.

Against this background, the present study aims to investigate the “ground zero” level for a specific university, to be taken into consideration when planning a sustainability plan for the student campus in the post-COVID-19 period, to identify the students’ potential to change their environment-related routines, due to EU policy changes and/or due to the university’s regulations, and to explore the possible directions for action towards increasing or contributing to the territorial sustainability in the socio-ecological context of Timisoara, the largest university city in the western part of Romania. Home confinement in 2020 and the first half of 2021 kept students away both from the physical academic facilities and, relevant for this study, from the campus. Therefore, the research team selected as topics that students could pinpoint from practice (and not from recollection) only those environment-related elements of consumption that they experienced in everyday life: water, plastic, and paper, leaving aside other elements such as mobility, clothing, or food consumption outside the household. The following research objectives are set for the study:

- (RO_1_) To determine the students’ water consumption routines (shower and drinking);
- (RO_2_) To identify the students’ environment-related routines with plastic and paper (avoiding waste generation and reducing plastic and paper consumption);
- (RO_3_) To investigate gender-bound preferences in environment-related routines in the household;
- (RO_4_) To identify the students’ potential to change their environment-related routines, due to EU policy changes and/or due to the university’s decision to go green in the post-pandemic period.

## 2. Literature Review

### 2.1. University as Part of the City’s Socio-Ecological System

The university is a constantly evolving institution, transformative for the generations of students, coming to seek knowledge and paths for their futures, but also capable of transforming itself, due to its tremendous, never-ending capacity to adapt to new levels of knowledge and societal expectations [23]. Despite the well-known metaphor of the “ivory tower” that is often applied to higher education institutions, throughout their history universities have always chosen to have some form of relationship with each other and with the city in which they are located, which led to the town vs. gown debate [24,25,26]. In the 21st century universities are seen as centers of learning and research, but also as agents of change—catalysts for social and political action towards sustainable development [10,24]. Their contribution to the sustainable development of the territory in which they operate is conducted both internally, by focusing on the curricula taught to students, and by ensuring that sustainability policies govern research, campus life and internal operations, and externally, via the university’s performance in the region [10]. There are voices that strongly speak for universities to step up to the role of being forerunners in facilitating the transition to a sustainable future [22]. In his seminal book, *Being a University*, Ronald Barnett bluntly states that “the time is such that the ecological university can be glimpsed, both as an idea and in its institutional form. The time is such also that the world needs its universities to be ecological” [23] (p. 141). However, his idea of ecology goes along the concerns regarding the university’s impact on the environment, and towards the philosophy behind the ecological project, an almost utopian enterprise [23,27].

Socio-ecological systems thinking, in which nature and society are understood as coupled and mutually produced, attracts the interest of an even larger audience [28]. Social-ecological systems are defined as “integrated complex adaptive systems in which social and ecological subsystems are coupled and interdependent, each a function of the other, expressed in a series of mutual feedback relationships” [29] (p. 3). The university fits into this interpretation, due to its continuous action toward creating links between generations of highly skilled intellectuals, the labor market, the regional and global arena, the ideal of the knowledge-based economy and the capability of providing models and guidance in promoting new solutions to the concerns of the society at large. On an action plan, many universities all over the world align themselves to the sustainability and the environmental aspect of their scholarship and operations through declarations, projects, and initiatives [15], the greening of universities being a phenomenon specific to the beginning of the 21st century [10], with various levels of success. As Renata Dagiliūtė states, the idea of green universities, so popular in Western practice, is less salient in post-socialist countries. In these countries, although ample transformative processes have taken place, higher education institutions wanting to join the green university model still have to invest massively in campus sustainability “by encompassing the programs of energy saving, waste management, food services, etc., especially behavioral shaping, that reaches students directly and enables them to act sustainably” [10].

In their role as institutions promoting societal change, being confronted with growing expectations regarding compelling contributions to sustainable changes, universities play a role also in educating young adults to contribute to sustainability goals, including the choices they make for their (future) households. Young people acquire “stuff” and organize their lives in a specific social and spatial–temporal context, thus reducing or increasing the environmental burden on their place of residence [30,31]. Especially as students, young adults face the thrills and challenges of becoming independent, “a process in life’s journey when practices are altered or become entrenched, for better or worse” [30]. Studies show that educational institutions (and governments) need to work towards reducing the gap between objective and subjective knowledge of environmental issues, so that students can make the right decisions based on their actual knowledge [32]. University-led initiatives toward the greening of institutional and the campus life are perceived as beneficial on multiple levels, such as offering vivid examples of social responsibility, saving money by reducing its resource consumption, or responding to the students’ activist spirit through events such as the Fridays for Futures climate strikes [15]. Ultimately, such initiatives contribute not only to demonstrating the university’s commitment to sustainability, but also to increasing the sustainability of the geographic territory in which they operate. Out of the many dimensions pertaining to the sustainable consumption, this study focuses only on water, plastic, and paper, as self-reported by students during the disruptive time of COVID-19.

### 2.2. Water Consumption

Besides the fact that the human body consists of 60–75% water and that a mere 15% loss of that amount can be fatal [33], water is necessary to prepare food, to wash, and to live in safe and healthy conditions.

The United Nation’s Renewable Development Goal 6 (SDG 6) aims at providing clean water supply for the world population by 2030 since billions of people are still deprived of clean drinking water. In other words, SDG 6 plans on ensuring the availability and sustainable management of water and sanitation for all people by 2030 in alignment with the fundamental human rights [34].

According to the SDG 6 indicators, in 2017, 2.2 billion people (i.e., 29% of the world’s population) did not have access to a safe drinking water source, 4.5 billion people did not have access to safe sanitation services and 3 billion people (i.e., 40% of the world population) did not have the possibility of washing themselves with water and soap at home [34].

Population growth and socio-economic development, as well as changes in consumption habits, have generated water stress. Water consumption has increased globally by approximately 1% a year since 1980, and it is estimated that, by 2050, it is going to increase by almost 20–30% over the current consumption level, with the domestic sector contributing to this trend as well [35].

**Figure A1:**
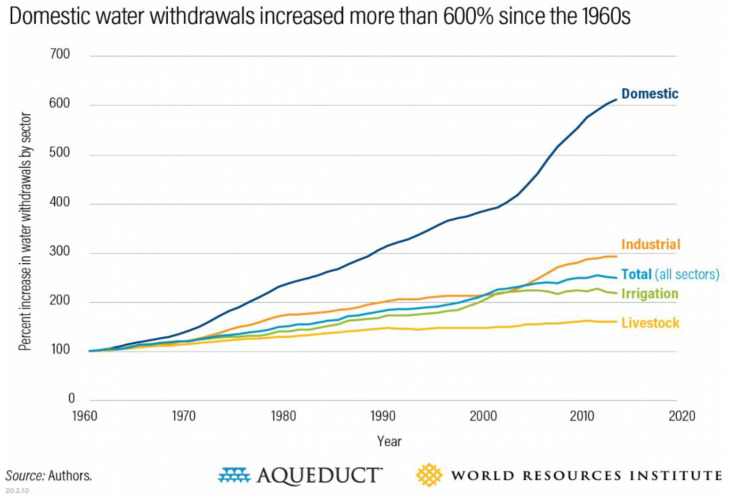
Global water use increase, 1960–2014. Source: https://www.wri.org/insights/domestic-water-use-grew-600-over-past-50 years (accessed on 17 June 2021).

Although, globally, the agricultural sector is still the biggest freshwater consumer, the statistics show that the water demand in the domestic sector has grown by 600%, at an accelerated pace, during 1960–2014 (Appendix A Figure A1) [36].

The European Union is no stranger to these problems, also being faced with concerns regarding water scarcity. Climate change (drought, less precipitation), pollution, urbanization, overconsumption, and mass tourism are the main contributors to water scarcity. Roughly 88.2% of Europe’s freshwater supply comes from rivers and groundwater, whereas the rest comes from reservoirs (10.3%) and lakes (1.5%), all these sources being extremely vulnerable to the above-mentioned threats [37].

In the General Comment Nr. 15, the UN’s Committee on Economic, Social and Cultural Rights defines the right to water as “sufficient, safe, acceptable, physically accessible and affordable water for personal and domestic uses”. The same comment stipulates the basic human need for water, pointing out that, “for personal and domestic use”, resources must include “drinking, personal sanitation, washing of clothes, food preparation, personal and household hygiene”. According to the World Health Organization, between 50 and 100 L of water per person, per day, are necessary to ensure basic water needs [38], which raises a serious question about how and if the needs of each individual can be covered at the current status quo.

**Figure A2:**
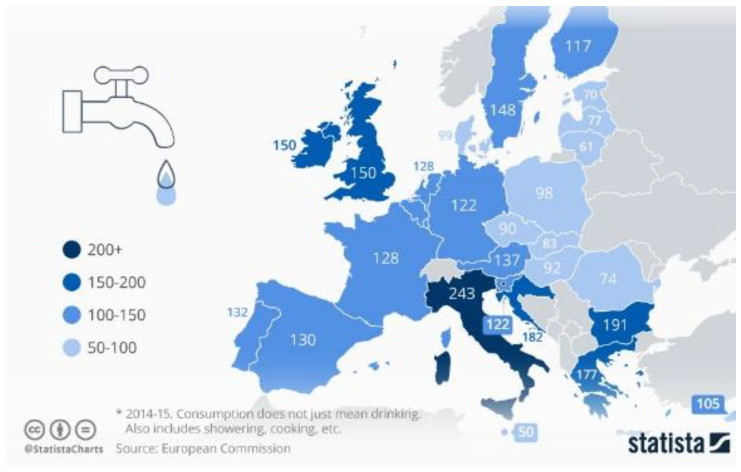
Average consumption of tap water per person in the EU, 2014–2015 (liters/day). Source: https://www.statista.com/chart/19591/average-consumption-of-tap-water-per-person-in-the-eu/ (accessed on 19 June 2021).

As far as the Europeans are concerned, the European Union data of 2016, for the period 2014–2015, show that the Italians used approximately 243 L per day, ranking first, whereas Romanians ranked last with only 74 L a day, based on data calculated by the providers of water services (Appendix A Figure A2) [39].

**Figure A3:**
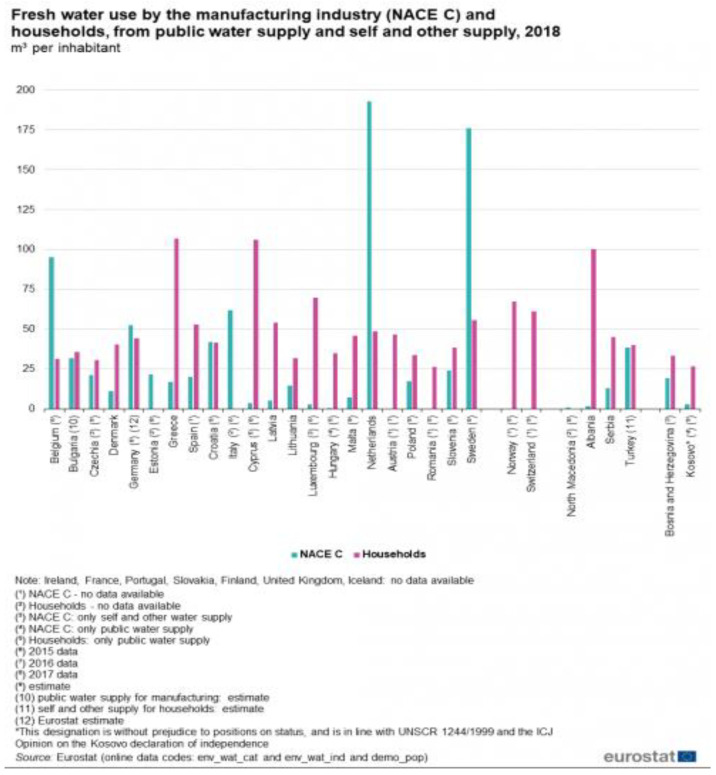
Drinking water consumption in the EU in 2018, m^3^ per inhabitant. Source: Water statistics—Statistics Explained (europa.eu) (accessed on 20 May 2021).

**Figure A4:**
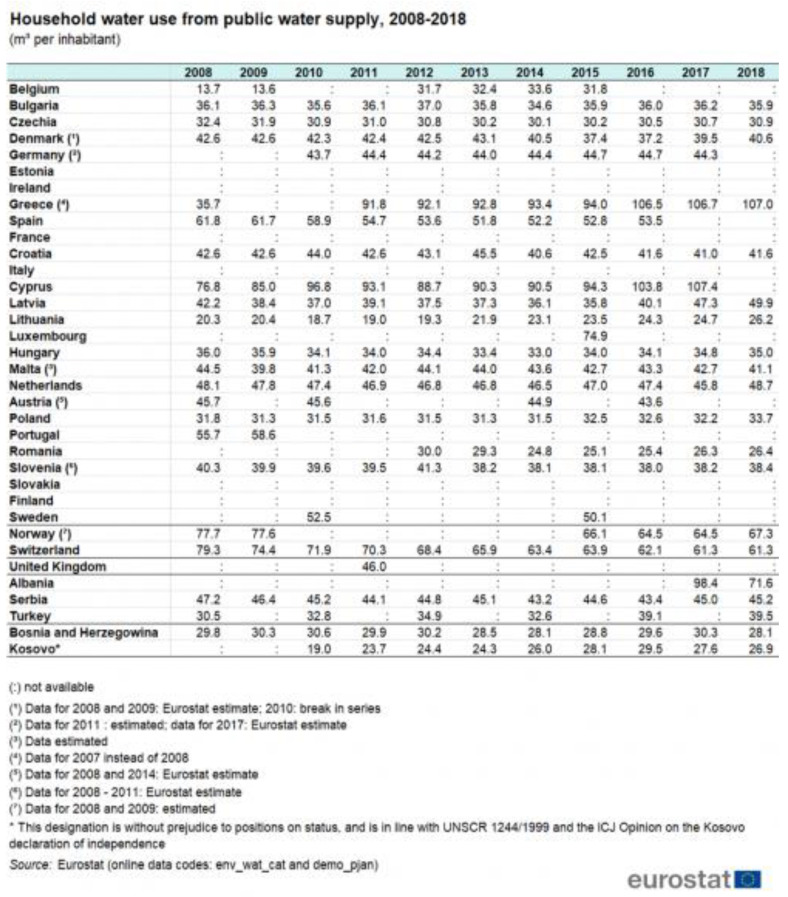
Household water use in the EU, 2008–2018, m^3^/inhabitant. Source: Water statistics—Statistics Explained (europa.eu) (accessed on 20 May 2021).

Romania also finds itself among the last countries in the 2018 ranking related to the drinking water consumption from public or individual sources within the EU, with 26 m^3^ per capita (Appendix A Figure A3) [40]. At the same time, a decrease in the drinking water use can also be noticed, from 30% in 2012 to 26.4% in 2018 (Appendix A Figure A4) [40].

The reason why water consumption is so low in Romania can be linked to the water price increase as a result of Romania’s alignment with the European directives, Romanian service providers being forced to increase their prices as a result of the investments carried out in the water treatment plants and of the works related to the extension of the water networks. The effect of the increase in water prices on household water consumption during 2002–2010 was studied by Ciomoș et al. [41].

**Figure A5:**
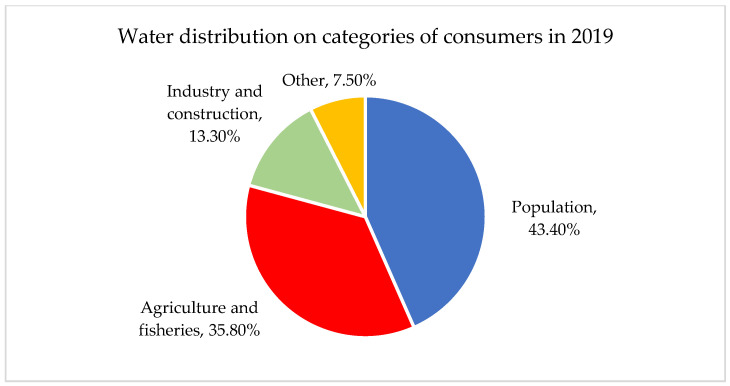
Water distribution based on categories of consumers in 2019, Romania. Source: https://insse.ro/cms/sites/default/files/com_presa/com_pdf/distributia_apei20r.pdf (accessed on 27 May 2021).

Another reason why, in Romania, water consumption is so low could also stem from the percentage of households that are connected to the public water supply. According to the National Institute of Statistics, the population connected to the public water supply system represented 70.9% (13,728,144 people) of the resident population in Romania in 2019 (19,370,448 people), which means that 5,642,304 people do not have access to the public water supply. The lowest percentage of households connected to the public water supply is in the north-east of Romania (50.1%), followed by Oltenia in the south-west (59.3%). The Bucharest–Ilfov region has the highest percentage of the population connected to the water supply (87.7%). At the same time, in 2019, the highest percentage of water (43.4%) was distributed to the population (Appendix A Figure A5) [42].

The reasons behind the different domestic water consumption behaviors are varied (the number of inhabitants, socio-economic factors, water saving devices, etc.) and have been studied by many researchers [43,44,45,46,47,48,49], some of them even suggesting social and economic models based on water consumption behaviors.

Corral-Verdugo et al. [43] devised a social model based on Mexicans’ water consumption behaviors. The authors noted that the perception that other people did not save water also reduced the other inhabitants’ desire to save water and led to increased water consumption; in other words, the citizens replicated others’ water consumption behaviors. Another social model, put forth by Gregory and Di Leo [44] and tested on Australian citizens, suggested that the annual water consumption was influenced by awareness of the environment, personal involvement, habits and other demographic characteristics (income, age, education, etc.) that influence water use (e.g., the use of a washing machine). An economic model, developed by Kenney et al. [47] on the inhabitants of Colorado, USA, showed that domestic water consumption depends on several parameters, such as price, weather as well as the restrictions imposed during severe climate conditions, such as drought.

Jorgensen. et al. [48] proposed an integrated socio-economic model, based on the already mentioned studies, which suggested that the demographic parameters, the household’s size, and the number of inhabitants have a direct influence on the water consumption. At the same time, the authors developed the Theory of Planned Behavior, stipulating that past behavior toward water consumption, trust in other persons (whether others save water or not) and in institutions, attitudes toward restrictions and the price of water during shortages influence the way in which people consume water.

Narrowing down the investigation to the students’ showering routines, British researchers highlighted the impact that administrative measures and persuasive campaigns had on the water consumption in Bristol [50], but the specificity of the local context makes it difficult to replicate the research in a different cultural and administrative context. The gap in researching students’ awareness of their water consumption habits, their attitudes toward saving water, or the issue of water management was highlighted in the scientific literature [51].

The above-mentioned studies do not fully succeed in explaining domestic water consumption based on the contextual parameters of the investigated populations. In Romania, just as in the case of electricity [8], there have been no studies to shed light on the domestic water consumption, be it sustainable or not. Such studies would be important since they could provide vital information for understanding a group of people’s attitudes towards water consumption in order for the authorities to develop a sustainable managerial plan and also in order to observe the necessity for educational resources on the sustainable water consumption.

Besides all these variables influencing the domestic water consumption, at the beginning of 2020, a new factor entered the equation, i.e., the lockdown following the COVID-19 pandemic. Therefore, because of schools closing and people working from home, water consumption began to increase in the households. In order to better their services for the population and to understand the rules of this new type of water consumption, the Water utility Stadtwerke Karlsruhe (SWKA) carried out an analysis on water consumption pattern changes in the city of Karlsruhe, Germany, which showed that the lockdown changes impact when and how much water people consume [52]. Even if official statistics regarding this topic are practically non-existent, researchers have studied these societal changes in different parts of the world, e.g., England [53,54], Germany [55], Uganda [56], Brazil [57]. In the case of Brazil, for instance, the showering routine increased by 32.4% during the lockdown, in comparison to the pre-COVID-19 period [58]. All these studies show a clear increase in domestic water consumption during lockdown and, thus, on people’s incomes, and were undertaken with the purpose of creating forecast models on household water consumption for better water supply during emergency times. Romanian studies on domestic water consumption during lockdown, as well as on its effects on the Romanians’ revenues have not been performed so far.

### 2.3. Paper and Plastic Use

**Figure A6:**
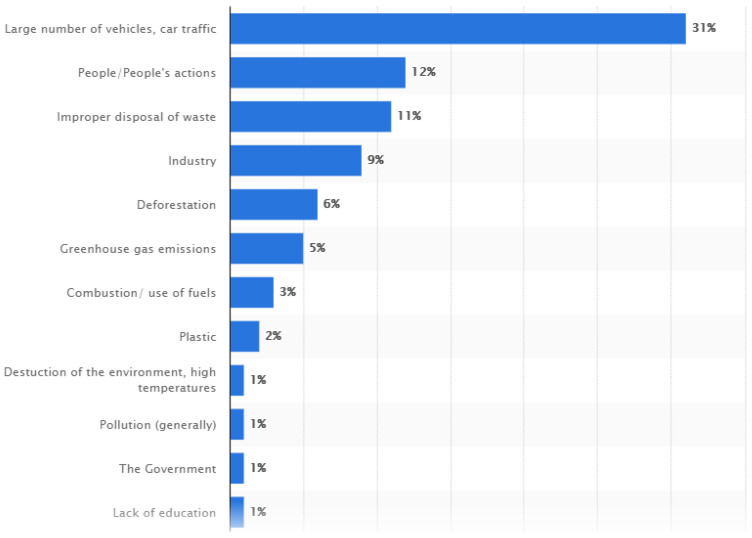
Main cause of global warming as perceived in Romania, 2019. Source: https://www.statista.com/statistics/1093956/perceived-causes-of-global-warming-romania/ (accessed on 8 June 2021).

**Figure A7:**
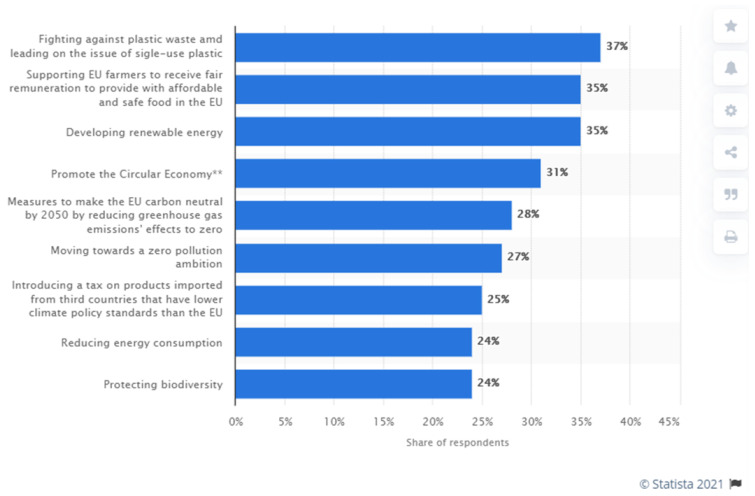
Romania: public opinion on the objectives in a European Green Deal 2019. Source: https://www.statista.com/statistics/1110123/european-green-deal-objectives-romania/ (accessed on 8 June 2021).

As has already been pointed out, the COVID-19 pandemic has severely impacted the routines of university students and may very well lead to permanent changes and new recommendations worldwide. In Romania, the context prior to the lockdown points to an acknowledgement of man’s impact on the environment, as results of surveys published by Statista [59,60] show that Romanians perceive as the main cause of global warming the increasing number of vehicles together with car traffic (31%), with the second most common responses being people’s actions (12%) and improper disposal of waste (11%), therefore setting the ground for changes in behavior and legislation (Appendix A Figure A6) [59]. Similarly to other Europeans, people in Romania seem to have a fair understanding of what can and cannot be achieved in the short term and opt to set realistic goals for sustainable development—such as combating plastic waste—rather than “aim of achieving zero pollution” (Appendix A Figure A7) [60].

It is worth noting that, since plastics first became popular more than half a century ago, the annual production has increased significantly. In 1950, the global plastic production amounted to 1.5 million metric tons. In comparison, 359 million metric tons were produced in 2018, of which 61.8 million metric tons were produced in Europe, for the packaging sector. Managing plastic waste is crucial to sustainable environmental planning, and even if Eurostat shows that the recycling rate of municipal waste has been rising constantly [61], it has to be noted that Romania (11.5%) is far behind other European countries such as Austria (58.2%), Slovenia (59.2%), or France (46.3%).

An interesting fact unveiled by the literature review is that the amount of plastic wastes generated worldwide since the outbreak has increased, and it is estimated at 1.6 million tons/day. Most of it was caused by medical needs, with approximately 3.4 billion single-use facemasks/face shields estimated to be discarded daily because of the COVID-19 pandemic, globally [62].

**Figure A8:**
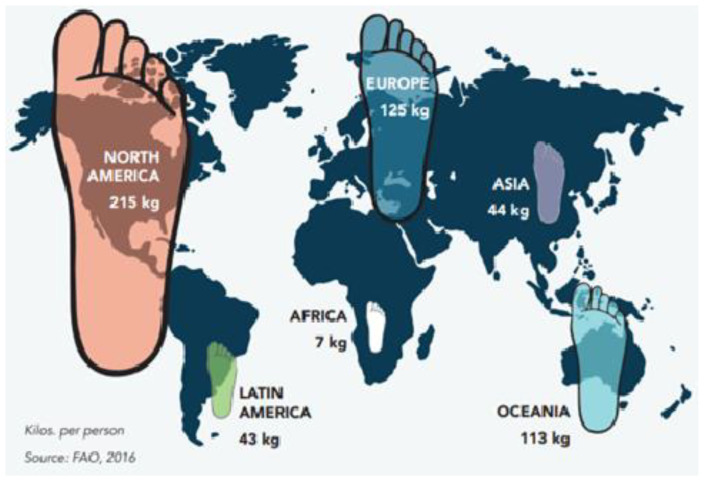
Per capita paper consumption, by region. Source: https://environmentalpaper.org/wp-content/uploads/2018/04/StateOfTheGlobalPaperIndustry2018_FullReport-Final-1.pdf (accessed on 8 June 2021).

**Figure A9:**
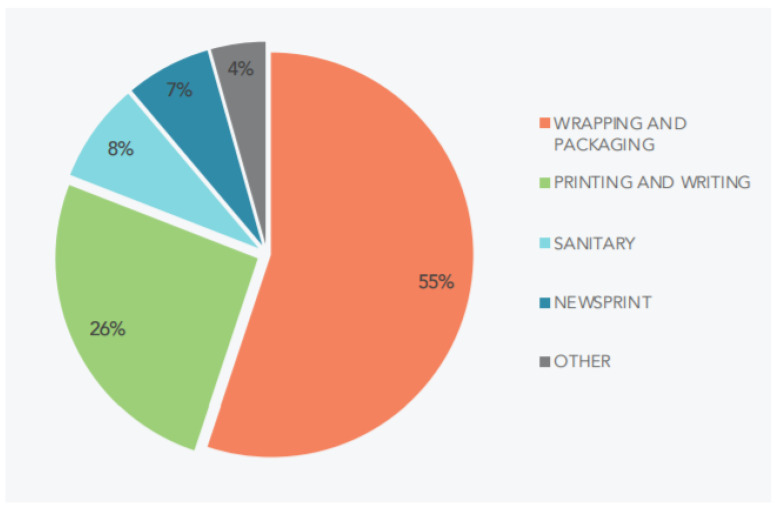
Global consumption by paper category, in tons. Source: https://environmentalpaper.org/wp-content/uploads/2018/04/StateOfTheGlobalPaperIndustry2018_FullReport-Final-1.pdf (accessed on 8 June 2021).

Similar information on paper waste management could not be found, as the majority of articles focus on estimating the changes that the COVID-19 outbreak has imposed on businesses [63,64]. Nevertheless, there is an abundance of data concerning global paper production and use, until 2019. For example, Appendix A Figure A8 [65] highlights the differences in paper consumption per region. The numbers are directly related to the current life habits of the inhabitants of the regions and are consistent with the findings of other research (Appendix A Figure A9) [65] which show that 55% of paper consumption is generated by wrapping and packaging, and 26% by printing and writing.

Taking into account the fact that most paper is produced from forestry products, usually trees, and that paper production is one of the largest polluters to air, water, and land, it becomes obvious why it is important to find solutions and develop environmental policies that will encourage the sustainable consumption of paper. These concerns, along with cost savings, are among the reasons for the shift towards going paperless, a trend in accordance with the needs of a globalized and digitalized world. On the other hand, cardboard is considered by many to be a comparatively renewable and sustainable option compared to plastic packaging, which is why we are witnessing a steady increase in the production of packaging paper and board.

However, if this paper were to be recycled, the positive impact on the environment would be enormous [66] because recycled paper production results in 40% fewer greenhouse gases, recycled paper requires 26% less energy to produce, and the production of the recycled paper creates 43% less wastewater.

One positive aspect of the COVID-19 home confinement could reside in the way educational institutions were obliged to adapt to a digital environment. For example, if a major constraint in reducing paper consumption was the fact that some lecturers provided hard-copy course documents and rejected electronically submitted assignments, preferring instead to have assignments submitted in the form of double-spaced or single-sided hard copies [67], the pandemic forced academics to adopt new recommendations and accept the context.

With increased awareness of these impacts and the rise and prevalence of digital technologies, there have been increased efforts among higher education institutions to go paperless [68]. While the paper industry works to minimize the impact of paper manufacturing, reducing human paper use from a consumer’s standpoint has its own set of unique challenges. Universities have worked to develop policies and incentives to decrease on-campus paper consumption including initiating printing quotas, restricting printing access for students, and requiring double-sided printing [69].

Even more, environmental sustainability has become a priority for many tertiary institutions and different initiatives have been formulated through declarations to foster sustainable development guidelines on how to incorporate sustainability into the university system [18,70].

## 3. Materials and Methods

### 3.1. Local Context

**Figure 1:**
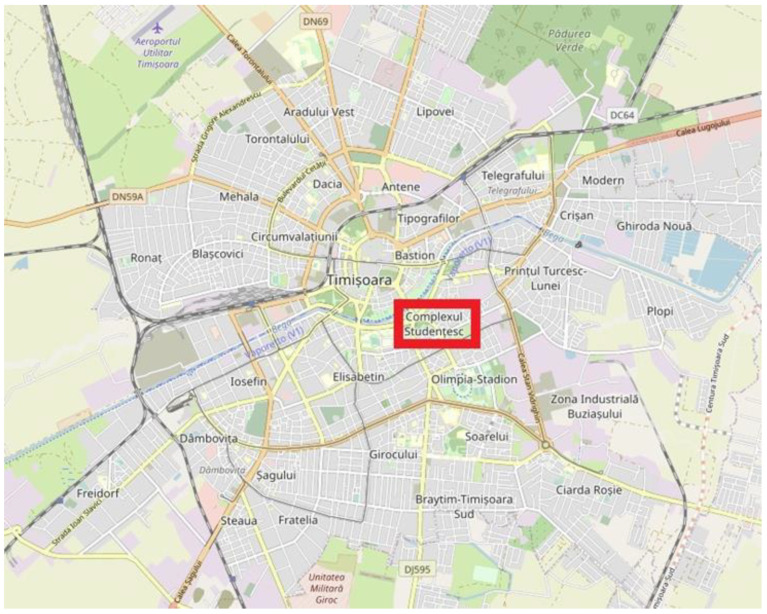
Map of Timisoara, with the student campus highlighted in the box.

Timisoara, the third largest city in Romania, became a university city in 1920, with the foundation of the (nowadays) Politehnica University. Currently, it is the educational choice for around 45,000 students, attending one of the four public universities or two private ones. Out of the total number of students, almost 40% are locals, and the rest come to the city during the academic year, which creates an increase in the number of residents of 10% from September through June, with short winter and spring breaks. The four public universities offer between them, institutionally, 13,000 places on the student campus (half of which belong to Politehnica University) (Figure 1), with the rest of the incoming students privately renting rooms or apartments in the city.

The outbreak of the COVID-19 pandemic in March 2020 sent the student population home and practically closed the campus, situated close to the city downtown area. According to local media, the local economy felt a loss of EUR 150 million due to the absence of students during the state of emergency and state of alert periods (2020—first half of 2021) [71]. The empty student campus, with almost zero consumption of municipal utilities and services, also impacted the overall administrative planning and activity during the period, this unprecedented crisis highlighting the ties between the city and the campus. On the other hand, even during the pandemic, Timisoara City Hall continued implementing, at the initiative of university-led projects, environment-related processes fitting into the European Green Deal [72], thus signaling its interest in promoting an active environmental policy for the development of the city and offering solutions to increase the sustainability of households and institutional residences. In its projections regarding the economic, social and environment planning for the city, Timisoara City Hall explicitly refers to UN Agenda 2030 for Sustainable Development and commits itself to take steps towards implementing SDG goals [73].

### 3.2. Sample

The study is a snapshot of students’ environment-related routines during the home confinement caused by the measures undertaken at state and local levels to contain the COVID-19 pandemic. No difference was made between students who live with their parents during their studies, those who resided on student campus in the pre-COVID-19 period or those who privately rent a residence during their studies. The survey was distributed to students of Politehnica University of Timisoara, the sample being one of convenience. In total, 816 subjects took part in the survey from all the study years. As the university’s student body counts around 13,000 students, the calculated margin of error is of ± 3.3%. Their average age, according to the recorded results, is 20.37 years old. Prior studies indicate that gender is a significant factor influencing environment-related choices in students [32]. The research team decided to investigate whether the gender variable is relevant for the Romanian case, and analyzed the sample through these lenses, with 409 female and 407 male subjects. Participation in the study was optional. No incentives were used to stimulate participation and students could opt out of the study at any time of the data collection. Additionally, no personal data were collected, to ensure students’ privacy and anonymous participation. The data were collected between March and April 2021.

### 3.3. Questionnaire

To collect the data, a non-standardized questionnaire was used, its content being validated through the following steps: assessment by experts (sociologists), followed by its qualitative and quantitative pretesting. To build the questionnaire, the everyday sustainable water, plastic, and paper consumption was referenced on specialized websites. Thus, a list of recommendations aimed at adopting sustainable behavior regarding the above-mentioned aspects was made. Finally, they were transformed into 5-point Likert-type scale questions that were included in the questionnaire.

The questionnaire was written in Romanian and was completed in 15 minutes on average. The statements were phrased in a neutral language, to collect students’ reflections on the matter without indicating the social desirability of environment-related routines, which could affect the reliability of the data. Additionally, the research team built the questionnaire having in mind the aim of reducing the social desirability biases, although the subjective character of responses is acknowledged [74].

Out of the three sustainable household elements of capability: (1) household practices (e.g., recycling and water conservation); (2) household structure (e.g., income, employment, dwelling type and composition); (3) household sustainability judgements (e.g., knowledge, awareness, concern towards climate change) [75], the research team focused only on the first one. For the purpose of the study, preeminence was given to the students’ environment-related routines, understood as familiar action patterns that involve regularity [76,77], and which are likely to be performed on a daily basis in the home confinement period caused by the COVID-19 pandemic.

### 3.4. Method

The instrument used for the data collection was the anonymous, self-administered online survey, posted on the Isondaje.ro platform (a Romanian free online survey service). The data were analyzed using SPSS (IBM SPSS Statistics variant 25).

At the end of their online classes, the students received from their teachers the link to the online questionnaire and the details needed to fill in the form. The average time required to answer the questionnaire was 15 min and the recorded response rate was of approximately 50%.

The analysis of the corpus of data relies on the self-reported information provided by the respondents and due to the variety of situations in the students’ housing arrangements (living alone, with peers, parents or grandparents, renting, in Timisoara or in other cities, etc.), they cannot be aggregated with actual data of their consumption of water, plastic, and paper, such a study being possible, at best, in the post-COVID 19 period, with an investigation of such parameters related to student campus life. To verify the gender-related difference in the obtained responses a chi-square Pearson test was applied.

## 4. Results

**Figure 2:**
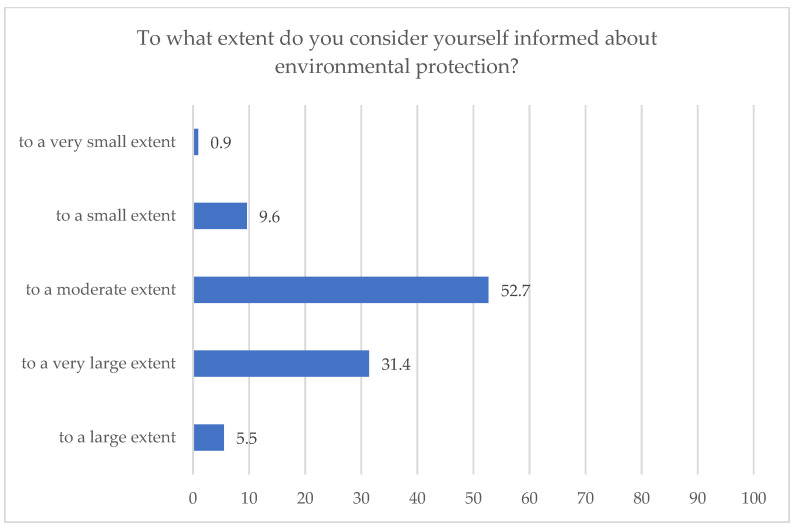
Respondents’ awareness of environmental protection.

A first point of interest of the study was finding out the level of awareness of the respondents regarding environmental protection. As can be noted in Figure 2, the highest percentages were recorded for “to a moderate extent” variant that was chosen by a little over half of the respondents (52.7%) and for “to a high extent” variant (31.4%). The percentage of those who consider themselves informed about environmental protection “to a small extent” and “to a very small extent” cumulates to 10.5%.

**Figure 3:**
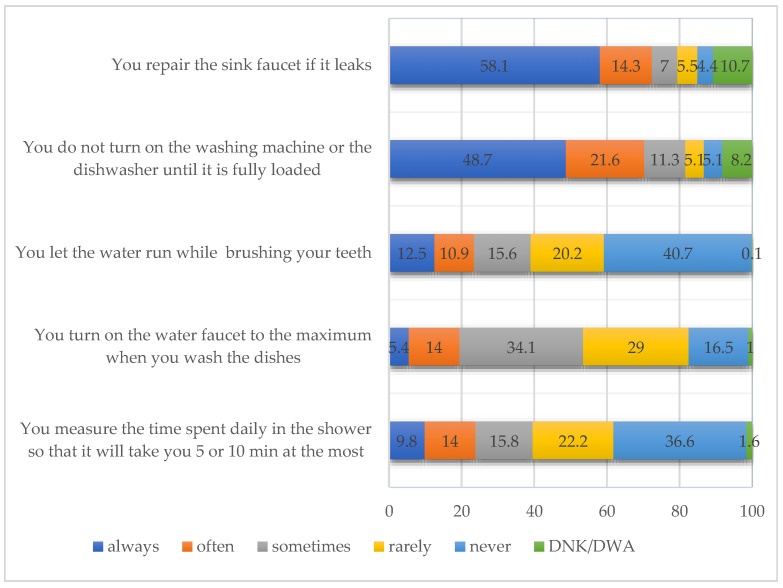
Respondents’ behavior towards water consumption in the household.

The next aspect researched by the study was determining the water consumption behaviors among students. Five sets of statements were created in the questionnaire, collected from specialized websites. For a better view and interpretation, the results were grouped according to the “often” and “always” as well as “rarely” and “never” response variants. The results can be seen in the figure below (Figure 3).

The statement that accumulated the highest percentage of responses and which shows that a sustainable water consumption behavior exists in households was “You repair the sink faucet if it leaks”. The “often” and “always” variants in this case cumulated 72.4%. “You do not turn on the washing machine or the dishwasher until it is fully loaded” cumulated a total of 70.3%. Although the statement “You let the water run while brushing your teeth” recorded a high percentage for the “rarely” and “never” variants (60.9%), they actually represent a positive and sustainable behavior regarding water consumption. The last variant is “Don’t know/Don’t want to answer” (DNK/DWA).

The same situation is recorded for “You turn on the water faucet to the maximum when you wash the dishes” where 45.6% of the responses suggest the absence of such a behavior. Still, in this case, this statement has recorded the highest percentage of responses for the “sometimes” variant, which leads to the presumption that the respondents’ behavior toward this household activity fluctuates.

An example of behavior that reveals the lack of awareness towards high water consumption has to do with personal hygiene and daily showers. “You measure the time spent daily in the shower so that it will take you 5 or 10 min at the most” recorded the highest percentages for the “rarely” and “never” variants, which together accumulated to 58.8%. In other words, it is likely that the level of awareness regarding the water waste in the household might be rather low.

A secondary analysis was carried out to verify whether there are significant differences between the female and male respondents. After applying the χ^2^ test, the results show that there are differences between males and females only in the case of the research statement ”You measure the time spent daily in the shower so that it will take you 5 or 10 min at the most”. For this statement a value of χ^2^ = 28.615 and a value of *p* = 0.00 (*p*< 0.05) were recorded. The results pinpoint the fact that there are notable differences based on gender, namely, that this type of behavior is more specific to females than to males.

**Figure 4:**
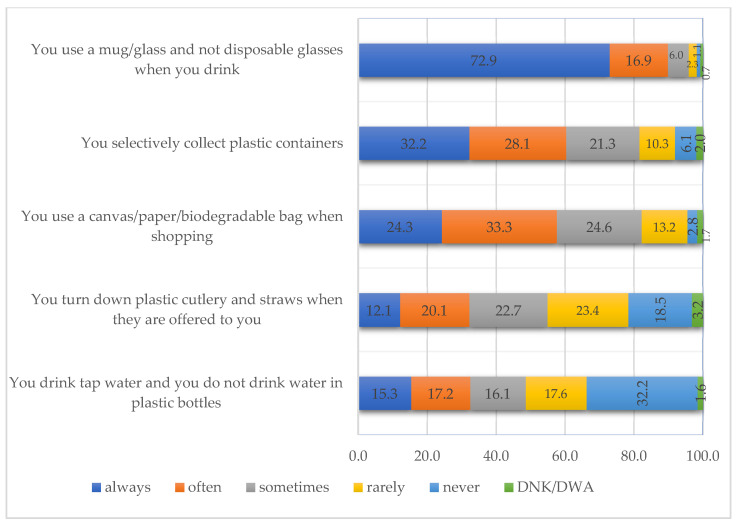
Respondents’ behavior towards plastic consumption in the household.

Another objective of this study was to capture the respondents’ behaviors regarding the use of plastic, and thus five statements referring to this aspect were included in the questionnaire. As can be seen in the figure below (Figure 4), the statement that accumulated the highest percentages for the “often” and “always” variants was “You use a mug/glass and not disposable glasses when you drink”. They scored 16.9% and 72.9%, respectively. Taking these results into account, it can be concluded that the respondents do not use plastic containers.

This statement is followed at quite a distance, as far as the percentages are concerned, by “You selectively collect plastic containers”, almost a third of the respondents declaring that they do this “often” (28.1%) and “always” (32.2%). By looking at the results obtained from those who answered “never” and “rarely” to the question, it can be observed that their percentage is relatively low, of 6.2% and 10.3%, respectively. “You use a canvas/paper/biodegradable bag when shopping” obtained almost a quarter of the responses for ”always” (24.3%) and almost a third for ”often” (33.3%). Therefore, as for the previous statements, this behavior can be found in more than half of the respondents.

At the other extreme, there are two statements for which the percentages obtained rather indicate the existence of a behavior of acceptance and use of plastic and not its refusal. “You turn down plastic cutlery and straws when they are offered to you” obtained a total score of 41.9% by cumulating the “rarely” and “never” response variants. This is the share of the respondents who accept their use. The last statement included in the study highlights again an acceptance of the use of plastic in everyday life. “You drink tap water and you do not drink water in plastic bottles” is the statement for which the highest values were recorded for the “never” variant (32.2%). The scores for the “rarely” variant, which obtained 17.6%, can be added to these values.

These statements were also checked to see whether significant differences between males and females exist. Following the application of the χ^2^ test, no significant differences related to the respondents’ gender were recorded. By analyzing the results obtained from the association tables, small differences in “You turn down plastic cutlery and straws when they are offered to you” can be noticed: female respondents accounted for 21.8% responses for “often” variant, while male respondents chose the same option only in 18.4% cases. For the “always” variant, female respondents chose the option in a proportion of 13.4% by comparison to male respondents with 10.8%.

**Figure 5:**
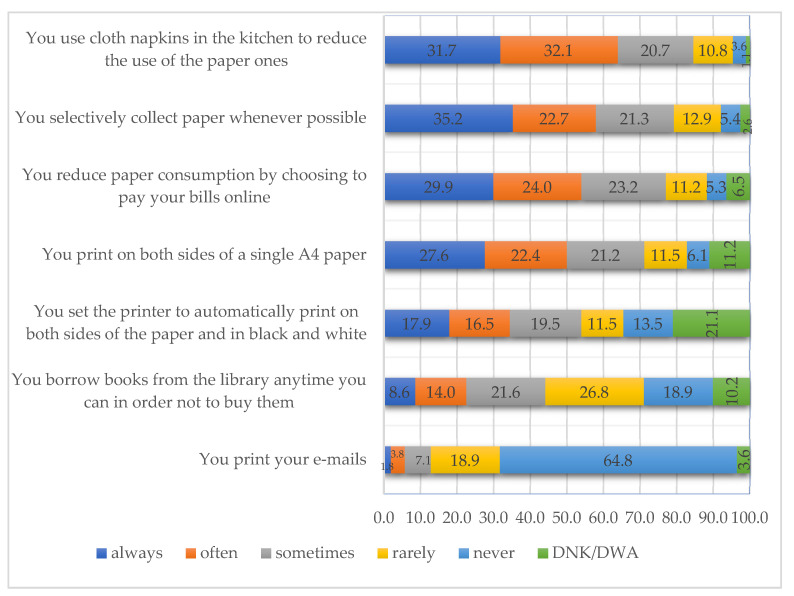
Respondents’ behavior towards paper consumption in the household.

The last objective of this study was to identify those behaviors that can lead to the avoidance of waste generation and to paper consumption reduction. As mentioned in the introductory part of this article, paper is one of the most common wastes in almost all fields of activity. Efficient paper management was captured in this study through the seven statements in the questionnaire targeting this behavior. To better view the results below, they were presented hierarchically, based on the results obtained for the “always” and “often” variants. As can be seen in the figure below (Figure 5), by accumulating the percentages for the above-mentioned variants, percentages higher than 50% were obtained for 3 of the 7 statements. Although “You print your e-mails” obtained the highest percentages for the variants situated at the other extreme, this result places it among the statements that lead to the idea of the existence of a behavior of avoidance of waste generation and of waste consumption reduction. By cumulating the results received for the “never” and “rarely” variants, a total of 83.7% is obtained, which confirms what was stated above. In the order of the percentages recorded for the variants that denote the existence of an ecological behavior, “You use cloth napkins in the kitchen to reduce the use of the paper ones” is the statement that follows, for which 63.8% of the respondents chose the “always” and ”often” variants (31.7% and 32.1%, respectively). The same happens for “You selectively collect paper whenever possible”, which accumulated values that exceeded 50% for the “always” and “often” variants (57.8%), being followed by “You reduce paper consumption by choosing to pay your bills online”, with a total of 53.9%.

“You print on both sides of a single A4 paper” scored exactly 50%. Lower percentages were obtained for “You set the printer to automatically print on both sides of the paper and in black and white” and “You borrow books from the library anytime you can in order not to buy them”, where the behavior of avoiding waste generation and of reducing paper consumption seems to be lacking.

These statements were also subjected to a second analysis to see whether there are significant differences between the male and the female population. Following the application of the χ^2^ test, differences between the two genders were identified for “You borrow books from the library anytime you can in order not to buy them”, which scored a value of χ^2^ = 35.182 and a value of *p* = 0.00 (*p* < 0.05). Therefore, these results show that there are significant differences related to gender in this case as this behavior seems to be more present in males and less present in females.

## 5. Discussion 

The survey enabled an evaluation of the environmental awareness of students at a given moment in time, in 2021, when home confinement due to measures undertaken to contain the COVID-19 pandemic limited their mobility and face-to-face interactions outside their households. The findings indicate a general inclination of respondents of practicing routines that spare resources (water and paper), control and reduce pollution (paper and plastic waste), resonating with those highlighted by Carducci et al. [78], who identified students as displaying sensitivity towards environmental issues. These authors also proposed that during the development of interventions to promote pro-environmental behaviors, the target population (i.e., students) should be studied including the health-related aspects, determinants, and obstacles in overcoming the gap between attitudes and behaviors in sustainability projects [78].

Research has shown that individual choices and lifestyles have a spillover effect and ultimately impact the environment, the natural resources, and the quality of (urban) living [11]. However, the complex socio-ecological system in which an individual evolves represents to a considerable extent the ‘sustenance base’ of their daily domestic (and work) routines [11]. The physical substratum to the domestic life is of consequence and in the case of environment-oriented individuals, striving for more sustainable lifestyles and patterns of domestic consumption, the possibilities existing in the socio-ecological systems are of strategic importance. Therefore, universities wanting to adopt a greening strategy for campus life need to understand the patterns of consumption specific for the generation that, after a period of home confinement, will return to the campus life and rejuvenates, at the same time, the city. The ecological modernization of production––consumption cycles will be easier to implement after the disruptive times of the COVID-19 pandemic and should aggregate the individuals affected by change (students), the university as a transformative institution, and also the city administration, as a provider of services that shape the domestic/living routines. Such environmental innovations can be accomplished via new techniques, but also via procedures, financial arrangements, etc. [11].

A sustainability-oriented public policy, such as the one declared by the Timisoara City Hall [72], should take into consideration not only the institutional efforts, but also the individual contributions to a better quality of life, a healthier environment, and a more prosperous economy. For this purpose, both at the individual and societal level, changes need to be made in the way resources are used, for their sustainable use and for the minimization of the environmental impact. The university campus can have significant effects on the environment both in terms of the resources it consumes and in terms of the problems it generates (pollution, waste, etc.). It should be the place where students, who represent an educated category of the population, can absorb the values related to sustainable development and can become the promoters of ecological behaviors both within their own households and at their future workplaces. It is a real laboratory where they can learn about sustainability, where models and opportunities can be created to change students’ behaviors, with the purpose of embracing values that can lead to societal transformation [15].

As mentioned above, this research does not aim at explaining students’ behaviors or at providing information about their triggers, but it is rather an assessment of these habits, capturing the reality during the COVID-19 pandemic. As D’Alessandro et al. rightfully state, “the ongoing pandemic of COVID-19 is a strong reminder that the lockdown period has changed the way that people and communities live, work, and interact” [79], and both outdoor and indoor spaces need to be re-examined from the point of view of their resilience and capacity to ensure the wellbeing of citizens. In the case of Romania, the absence of such studies at a local, regional, and even national level and the lack of information regarding the way water, plastic and paper are consumed are solid arguments in favor of the need for this analysis. Even though the study was carried out on a distinct category of respondents, the students of Politehnica University of Timisoara, these results are useful in planning policies that could lead to the adoption of sustainable behaviors both by this category of consumers and by other categories of the population as well. They are also necessary to carry out campaigns meant to inform the population about sustainable consumption, which cannot be conducted without being first aware of these habits. Previous research warns that tailoring information and persuasion campaigns can prove not to be cost-effective in many cases and that at different levels of aggregation (such as the community, municipal level, regional, or even country levels) a thorough knowledge of the differentials in the target population is necessary [80].

One aspect that can be drawn from the results of the study is that awareness campaigns should be conducted among students regarding general environmental problems, since about half of them say that they are informed about them “to a moderate extent” and about a third of them consider themselves informed of such problems to a large extent and “to a very large extent”.

As far as water consumption is concerned, for more than half of the respondents, habits that show the existence of sustainable consumption behaviors, such as “You repair the sink faucet if it leaks”, “You do not turn on the washing machine or the dishwasher until it is fully loaded” and “You do not let the water run while brushing your teeth”, have been identified.

Another aspect highlighted by the study is the fact that actions should be taken primarily to raise awareness of the high water consumption in daily showers, a situation in which more than half of the respondents said that they do not “measure the time spent daily in the shower so that it will take them 5 or 10 min at the most” and in kitchen activities when they “turn on the water faucet to the maximum when they wash the dishes”. The secondary analysis carried out on the corpus has also pinpointed a notable difference based on gender, i.e., a certain type of water consumption behavior is more specific to females than to males, for the above statement, resonating with findings in previous research [32]. However, although the presence of sustainable consumption behaviors has been identified for more than half of the respondents, there is still a significant share of the student population that still needs to be made aware of these problems and of these behaviors. Additionally, the fact that the results resonate with the findings of Vicente-Molina that gender roles might be decreasing in importance in some environmental tasks, especially in the student population, is an issue that requires an in-depth analysis [32].

Awareness campaigns for sustainable water consumption could be carried out by involving student organizations, which can act as “pipelines” through which information could be passed on to the students. These campaigns could also be carried out by involving the university management to identify some technical measures to optimize water consumption on campus (water faucet timers, economical shower heads, dual toilet tanks, etc.) and the dormitory management to help implement these solutions [50]. Another possibility is to introduce disciplines or new chapters in the existing curricula that target sustainable development. Finally, environmental associations, water suppliers and local authorities could also be involved; through mutual efforts, they could create a snowball effect with significant results in embracing sustainable water consumption behaviors. Studies show that when pro-active measures are undertaken, students respond positively, reflect on the possibility of adopting sustainable patterns of resource consumption and feel empowered to act responsively on campus [81].

Reducing the negative impact of plastic on human health and on the environment can be achieved by limiting its everyday use through reuse, or selective collection with the purpose of recycling. Analyzing students’ environment-related routines has revealed interesting aspects regarding plastic consumption, which may indicate that further efforts should be made to educate students on how to reduce their carbon footprint. For example, the results showed that the majority of students selectively collect plastic containers (60.3%) and use “a canvas/paper/biodegradable bag when shopping” (57.6%), but 41.9% of them do not “turn down plastic cutlery and straws when they are offered” to them. Behavioral differences based on gender were merely evident, with green-oriented behavior being slightly more present in female respondents than in male respondents.

As mentioned in the introductory part of this study, paper is one of the most common wastes in almost all fields of activity, therefore understanding students’ routines was key to evaluating the needs of future in-campus policies. The results lead to the idea of the existence of a behavior of avoidance of waste generation and of waste consumption reduction. A cumulated percent of 83.7% of students “never” or “rarely” print their emails, 57.8% of them “selectively collect paper whenever possible”, and 53.9% “reduce paper consumption by choosing to pay your bills online”. On the other hand, printing on both sides of the paper and in black and white is not yet the prevalent behavior. The research also identified a significant gender-based difference in the case of the statement “you borrow books from the library anytime you can in order not to buy them”, this behavior being more present in males than in female students.

The sustainable household capability is an achievement, the outcome of a specific social and cultural context that offers sufficient possibilities to reduce emissions, as Gordon Waitt et al. convincingly demonstrate [75]. According to these authors, “some households may accept the environmental science that frames sustainability issues, including climate change, but their everyday social contexts, habits and routines may render pro-sustainability actions as unthinkable” [75]. As D’Alessandro et al. also convincingly point out, to increase the sustainability and resilience of households it is necessary to “pool the knowledge from the technical field and Public Health expertise”, to identify exportable and scalable best practices and engage multisectoral responsibility, in order to overcome the vulnerabilities highlighted by the COVID-19 pandemic [79]. In the case of a university campus, as this study proposes, there are sufficient tiers of interventions that the university, jointly with the municipal administration, can undertake to encourage more vigorous pro-environmental behaviors, to increase the sustainability of the campus and, due to the spillover effect, of the administrative territory where the campus is operated.

The COVID-19 pandemic has obliged students and teachers to adapt to a virtual environment and find solutions to continue the educational process under new conditions. Therefore, with respect to environmental protection and sustainable development, the quick changes that had to be implemented can be considered a big step forward and universities could incorporate efforts toward resource preservation and the encouragement of green behavior as part of their recovery plan from the disruptive times of the pandemic, responding in a pro-active manner to the calls for a greener, more sustainable higher education [5,6,7]. Such actions can be implemented by all European universities, along the path already opened by the Green University model [15], with the cautious selection of measures that Renata Dagiliūtė et al. [10] stated for the post-socialist bloc that includes Romania. The present study focuses on a university that is perceived by its students as “trustworthy, effective, modern”, and thus capable of leading the way for implementing innovation [82]. It is, therefore, to be expected that a greening action would be welcomed by students. Since students represent a quarter of all decision-making bodies in the university, their informed action is a precondition for all initiatives, environment-related ones included.

## 6. Limitations and Future Extension of the Study

The main limitation of this study acknowledged by the research team is that the results cannot be extrapolated to the whole student population in Timisoara, to other university students in Romania or to other geographical contexts. Researchers have shown that environmental choices are culture and context-bound [22,32]. These findings also need to be compared with data on students with a variety of contextual and situational factors (e.g., students who are working and studying simultaneously, who live with their parents or rent independently, outside the university campus, or chose to stay on campus for their studies and work). Such factors were not relevant during the COVID-19 pandemic, with students being practically home-confined for more than a year, but they need to be accounted for in future studies. Another limitation is that the study is based on self-reporting, and it would be useful to replicate the current research with objective behavior measures and aggregate the findings with data from relevant authorities (campus administration and/or municipal services). The broader survey methodology literature [32] highlights that inaccuracy in the results may stem from the subjective interpretation of the respondents, who attribute different meanings to the issues raised by the questionnaire or who, under the influence of the topic of the survey, attempt to overreport their pro-environmental behavior. Additionally, the research should be expanded with more survey instruments to model latent factors and measure variables such as motivations/value orientations, and environmental concern, which other researchers have considered to be influential.

The initial results are being used to help develop targeted strategies for minimizing water, plastic and paper consumption on campus and will be incorporated into the university’s zero waste management plan that is currently under development. The effectiveness of such measures, the practical aspects of implementation and the resonance of this university-led initiative are also of research interest, especially since they are correlated with the larger context of the socio-ecological urban system and with the efforts undertaken by administrative bodies to increase Timisoara’s sustainable feature.

## 7. Conclusions

The findings in the present study have theoretical, practical and managerial consequences, as well as policy implications. From a theoretical point of view, this research contributes to the body of knowledge regarding young people’s behavior during emergency times when many of the habitual choices are canceled. While research on crises is extensive and well-documented, the COVID-19 pandemic puts its specific imprint and requires distinctive attention, with effects still to be evaluated when the health issued are solved. 

Research on routines related to sustainable consumption should help practitioners understand how to motivate individuals (in this case—students, young, highly skilled adults) to engage in more sustainable consumption. From the perspective of institutions (universities) and administrative bodies, being confronted with sustainability expectations and/or trying to incorporate sustainability arguments for shaping more resilient policies, it is important to know which foci regarding the sustainability dimensions [12] resonate with the various segments of population, the student body in the case of Timisoara representing 10% of the city’s residents. Insufficient motivation, environmental knowledge, and the lack of physical availability of sustainable solutions might be detrimental to pro-environmental behaviors [32]. Educational institutions, local administrations and governments should attempt to reduce the gap between objective and subjective knowledge of environmental issues, so that students can make informed decisions based on their actual knowledge and practice and feel empowered to act responsively on and off-campus.

Therefore, universities wanting to adopt a greening strategy for campus life need to focus on developing awareness campaigns, which, in accordance with the findings of other studies [10,13,50,51,68,78], is crucial to the success of implementing sustainable consumption habits. Universities alone cannot implement extensive greening measures without coordinating their efforts with the utility services providers (water services, waste services, etc.) and, further, with the authorities in charge of organizing such services. At the specific level of Timisoara, which has been analyzed in this study, the political will is present as expressed in the city administration’s program that makes reference to the European Green Deal. The appropriate policy measures, however, still have to be undertaken. The timing is of essence and the disruption brought about by the COVID-19 pandemic can become the needed opportunity for universities, as transformative institutions, to be vectors of change.

## Data Availability

The data presented in this study are available on request from the corresponding author.
